# Stochastic Resonance with Colored Noise for Neural Signal Detection

**DOI:** 10.1371/journal.pone.0091345

**Published:** 2014-03-14

**Authors:** Fabing Duan, François Chapeau-Blondeau, Derek Abbott

**Affiliations:** 1 Institute of Complexity Science, Qingdao University, Qingdao, P. R. China; 2 Laboratoire d'Ingénierie des Systèmes Automatisés, Université d'Angers, Angers, France; 3 Centre for Biomedical Engineering and School of Electrical & Electronic Engineering, The University of Adelaide, Adelaide, Southern Australia, Australia; McGill University, Canada

## Abstract

We analyze signal detection with nonlinear test statistics in the presence of colored noise. In the limits of small signal and weak noise correlation, the optimal test statistic and its performance are derived under general conditions, especially concerning the type of noise. We also analyze, for a threshold nonlinearity–a key component of a neural model, the conditions for noise-enhanced performance, establishing that colored noise is superior to white noise for detection. For a parallel array of nonlinear elements, approximating neurons, we demonstrate even broader conditions allowing noise-enhanced detection, via a form of suprathreshold stochastic resonance.

## Introduction

Stochastic resonance has emerged as a significant statistical phenomenon where the presence of noise is beneficial for signal and information processing in both man-made and natural systems [1]–[11]. The excitable FitzHugh–Nagumo (FHN) neuron model has been discussed for exploring the functional role of noise in neural coding of sensory information [12]. Following this, the milestone concept of aperiodic stochastic resonance using the FHN neuron model [13] stimulated a number of interesting investigations in sensory biology [3], [7], [14], [15] and physiological experiments [6], [8], [16]–[20]. Due to the character of activity in the nervous system, the neuron coding strategy based on stochastic resonance is also found in threshold (level-crossing) [21]–[23] and threshold-free [24]–[28] neurons. Since there are large numbers of neurons in the nervous system of animals and humans with variations in structure, function and size [2], [4], [7]–[9], then the potential exploitation of stochastic resonance in a neuron bundle becomes an interesting open question in neuroscience. In a general summing neural network, Collins *et al*. [4] reported that the noise intrinsic to each neuron could be used to extend the operating range of the sensitivity of the overall system. This, however, is not a unique case. In the summing multi-threshold network, suprathreshold stochastic resonance discovered by Stocks [29] overcomes the restriction of subthreshold signals, and appears to offer a possible explantation of dc adaptation in sensory neurons [9], [30]. One-dimensional coupling [31] and spatio-temporal stochastic resonance [7], [32] show that not only an optimal noise intensity but also an optimal coupling strength exists. Recent stochastic resonance research in complex networks [33]–[40] also demonstrates that an interconnected network configuration, as well as the non-zero noise level, can be optimized to achieve the best system performance.

In many practical situations, the idealization of white noise is never exactly realized [2], [5]. Consequently, the effect of colored noise on stochastic resonance has been investigated using the measure of output signal-to-noise ratio of a periodic signal [2], [5], [16], [41]–[43]. Although the suppression of stochastic resonance with increasing noise correlation time was demonstrated [2], [5], [41]–[43], it is interesting to note that, under certain circumstances, colored noise can be superior to white noise for enhancing the response of a nonlinear system to a weak signal [16], [44]. In the field of signal detection, the employment of noise to enhance signal detectability also becomes a possible option [45]–[55]. However, in most of these studies, the observed noise samples are often assumed to be independent. Colored noise for signal detection [56]–[60] is not adequately investigated in the context of stochastic resonance. In this article, we focus on the weak signal detection problem with the beneficial role of additive colored noise in threshold neurons. Because of the “all-and-none” character of nerve activity [61], the problem of threshold-based neural signal detection can be considered as a statistical binary hypothesis test [7], [23], [27]. In this situation, explicit expressions for the maximum asymptotic detection efficacy are derived for a given transfer function of neuron model. We prove that colored noise that arises from a moving-average model is superior to white noise in improving the detection efficacy of neurons. It is illustratively shown that, for a single neuron with a signum threshold nonlinearity, the possibility of noise-enhanced detection only holds for non-scaled noise. For scaled noise, the effect of noise-enhanced detection does not occur in a single neuron model. However, when we tune the internal noise components of a parallel array of threshold neurons, it is observed that the constructive role of noise comes into play again in improving the signal detection efficacy, wherein suprathreshold stochastic resonance manifests its potentiality.

## Results

### Detection model

Consider the detection problem formulated as a binary hypothesis test [23], [60], [62]

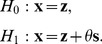
(1)Under hypothesis 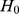, the observation vector 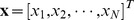 consists of noise 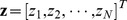 only, and under hypothesis 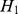 it consists of noise 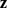 and known signal 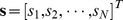 with its strength 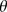. There exists a finite bound 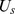 such that 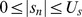, and the asymptotic average signal power satisfies 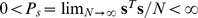 [56]–[58], [60], [62]. Next, the test statistic 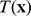 is compared with a decision threshold 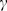 to decide the hypotheses, as
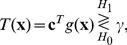
(2)where the coefficient vector 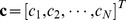 is associated with the function 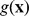 to form 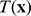.

Assume the 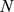-dimensional probability distribution 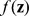 of noise 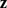 and zero-mean vector of 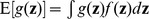 (for a shift in mean) [58], [60]. Then, for a large sample size 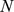 of observation vector 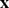, the test statistic 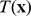 has zero-mean and asymptotic variance
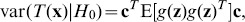
(3)under hypothesis 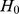. Furthermore, for weak signals (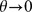) and under hypothesis 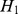, 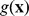 can be expanded to the first-order
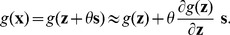
(4)Then, the characteristics of 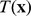 under 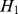, up to the first-order in 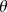, can be calculated as
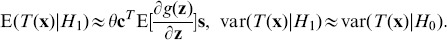
(5)Under both hypotheses 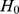 and 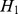, the test statistic 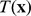, according to the central limit theorem, converges to a Gaussian distribution. Thus, the binary hypothesis test of Eq. (1) becomes a Gaussian mean-shift detection problem [60], [62]. Given the false probability, the detection probability is a monotonically increasing function of the detection efficacy 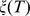 [60], [62] given by
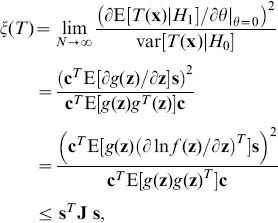
(6)where the Cauchy-Schwarz inequality yields
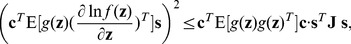
(7)with the Fisher information matrix 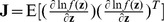. Note that the equality of Eq. (6) is satisfied by the locally optimum nonlinearity
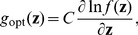
(8)for an arbitrary constant 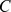.

However, a complete closed-form noise distribution 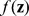 may be unavailable, especially in unknown noisy circumstances [56]–[58], [60], [62], which makes the nonlinearity of Eq. (8) difficult or too complex to implement. Thus, there may be compelling reasons for considering the given function 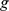 with an easily implemented feature. In this case, the detection efficacy in Eq. (6) can be maximized as
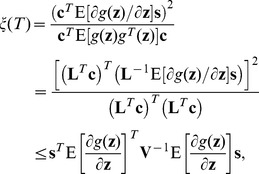
(9)with the Cholesky decomposition of the variance matrix 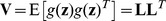 and by optimally choosing the coefficient vector 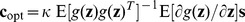 for an arbitrary constant 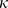.

### Colored noise

**Figure 1:**
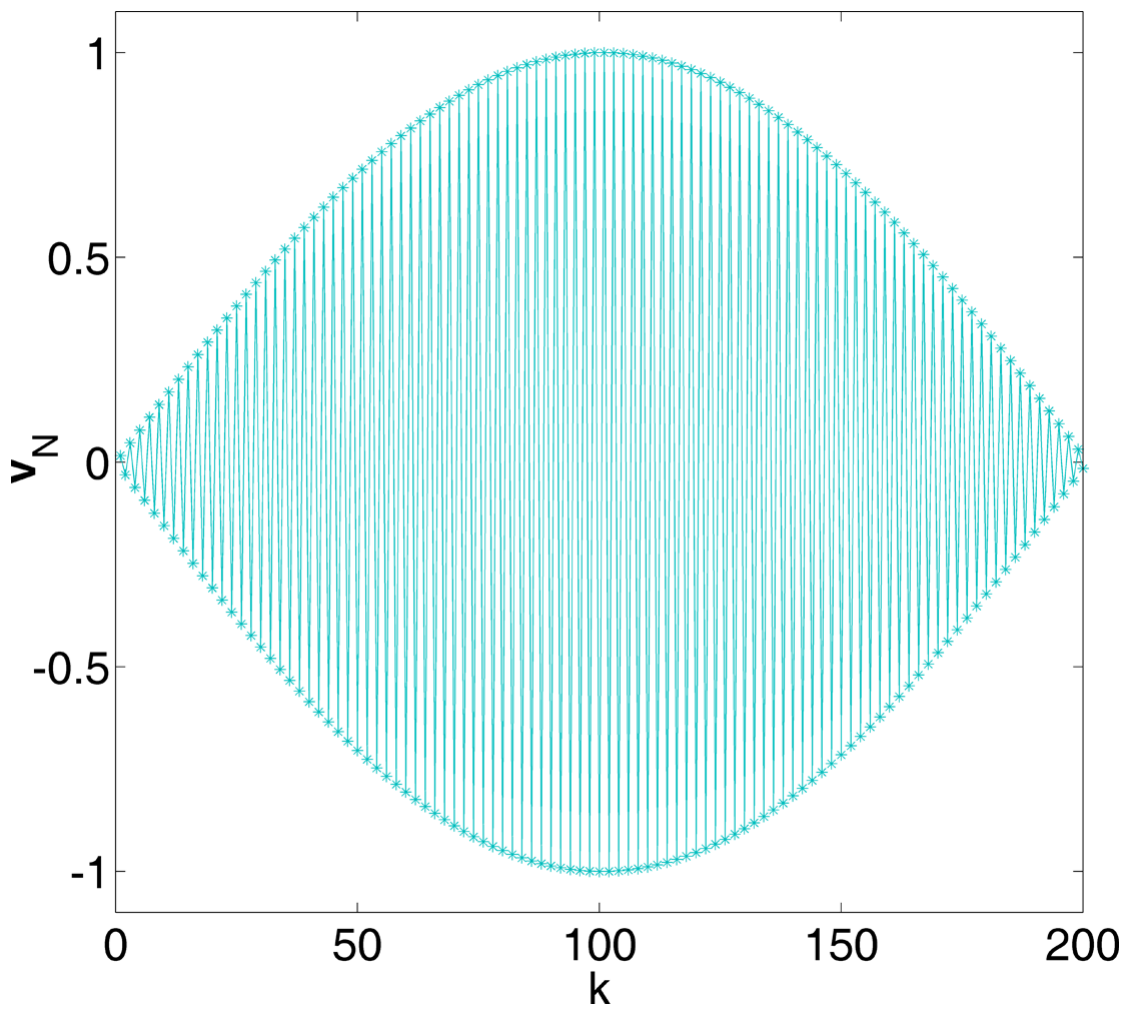
Eigenvector 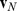. An illustration of the eigenvector 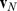 of the variance matrix 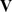 (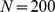).

Consider a useful colored noise model of the first-order moving-average [56], [59] as
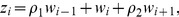
(10)where the correlation coefficients are 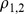 and 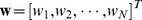 is an independent identically distributed (i.i.d.) random vector. For small values of 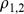 (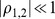), the dependence among noise samples 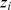 will be weak [56], [59]. Here, we assume 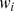 have an univariate distribution 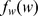 that is symmetric about the origin. We also assume the memoryless nonlinearity 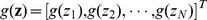 to be odd symmetric about the origin. Then, up to first order in small correlation coefficients 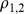, we can expand 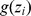 as
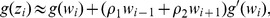
(11)

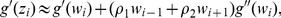
(12)and obtain expectations
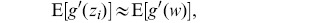
(13)

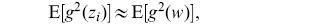
(14)

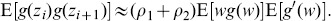
(15)Therefore, we have the expectation matrix
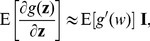
(16)with the unit matrix 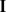, and the variance matrix 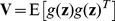 has elements
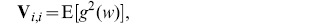
(17)

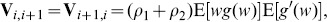
(18)for 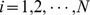. Then, based on Eq. (9), the normalized detection efficacy 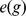 can be computed as
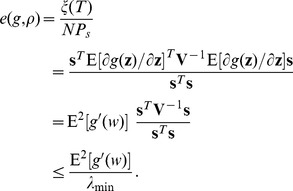
(19)Here, when the equality of Eq. (19) is achieved, the signal 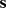 is the corresponding eigenvector to the minimum eigenvalue 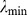 of the matrix 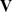. It is known that the eigenvalues of the matrix 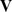 are [62]

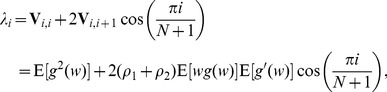
(20)corresponding to eigenvectors 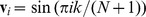 for 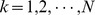. Here, as the nonlinearity 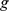 is assumed to be odd, it is then found that 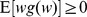 and 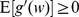. Therefore, if 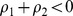 and for a large sample size 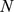, we take 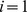 and 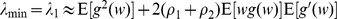. Otherwise, we choose 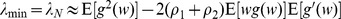. An illustration of the eigenvector 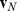 is shown in Fig. 1 for 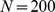. In this way, by optimally choosing the input signal (eigenvector) 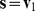 (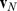), the maximum efficacy 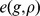 can be calculated as
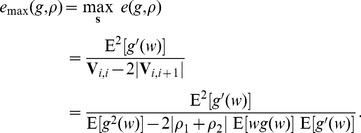
(21)


Since, from its definition in Eq. (6), the efficacy 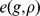 is non-negative, the denominator in Eq. (21) must satisfy
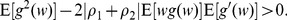
(22)In order to validate Eq. (22), we use the Cauchy-Schwarz inequality to yield
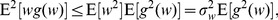
(23)

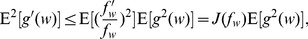
(24)with the Fisher information quantity 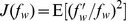 and the variance 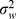 of noise distribution of 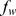 [56]. Thus, we find
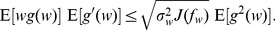
(25)Substituting Eq. (25) into Eq. (22) and noting
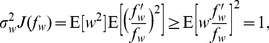
(26)we have
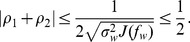
(27)Since we assume 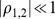, the inequalities of Eqs. (27) and (22) can be satisfied, and the detector efficacy in Eq. (21) will be theoretically analyzed in the following.

For white noise vector 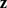 with zero correlation coefficients 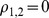, the detection efficacy 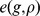 in Eq. (21) satisfies
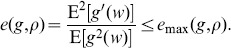
(28)Thus, for a given function 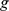, colored noise is superior to white noise in enhancing the detection efficacy, at a cost of optimally matching the input signal with the eigenvector 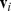 of covariance matrix 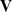.

### Stochastic resonance in threshold-based neurons

We will illustratively show the possibilities of noise-enhanced detection in threshold-based neurons. The classical McCulloch-Pitts threshold neuron has the form
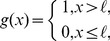
(29)with the response threshold 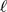. It is seen that 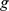 can be expressed as a function of 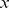 in terms of the signum (sign) function as 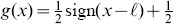. Since the constant factor 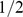 does not affect the detection efficacy of the transfer function 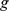, then we focus on the signum function
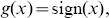
(30)with response threshold 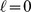 in the following parts. Here, the signum function 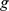 is not continuous at 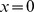, but has its derivative 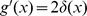 for any 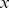 [60].

For the colored noise model of Eq. (10), the correlation coefficient 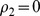 indicates the noise sequence 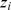 is a causal process that can be physically realized. Here, we assume 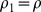 (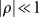) and 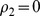, and show the possibility of stochastic resonance in the physically realizable noise environment. First, consider scaled noise 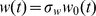 that has the distribution 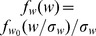 [23], [60]. Here, 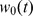 has a standardized distribution 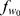 with unity variance 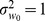. Thus, based on Eq. (21), the absolute moment is
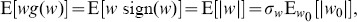
(31)where the operator 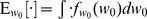. Thus, for the signum function 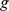, the detection efficacy of Eq. (21) can be expressed as
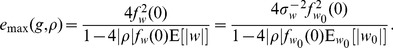
(32)It is seen in Eq. (32) that 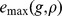 is a monotonically decreasing function of noise variance 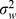, and no noise-enhanced detection effect will occur in such a single neuron model for scaled noise.

**Figure 2:**
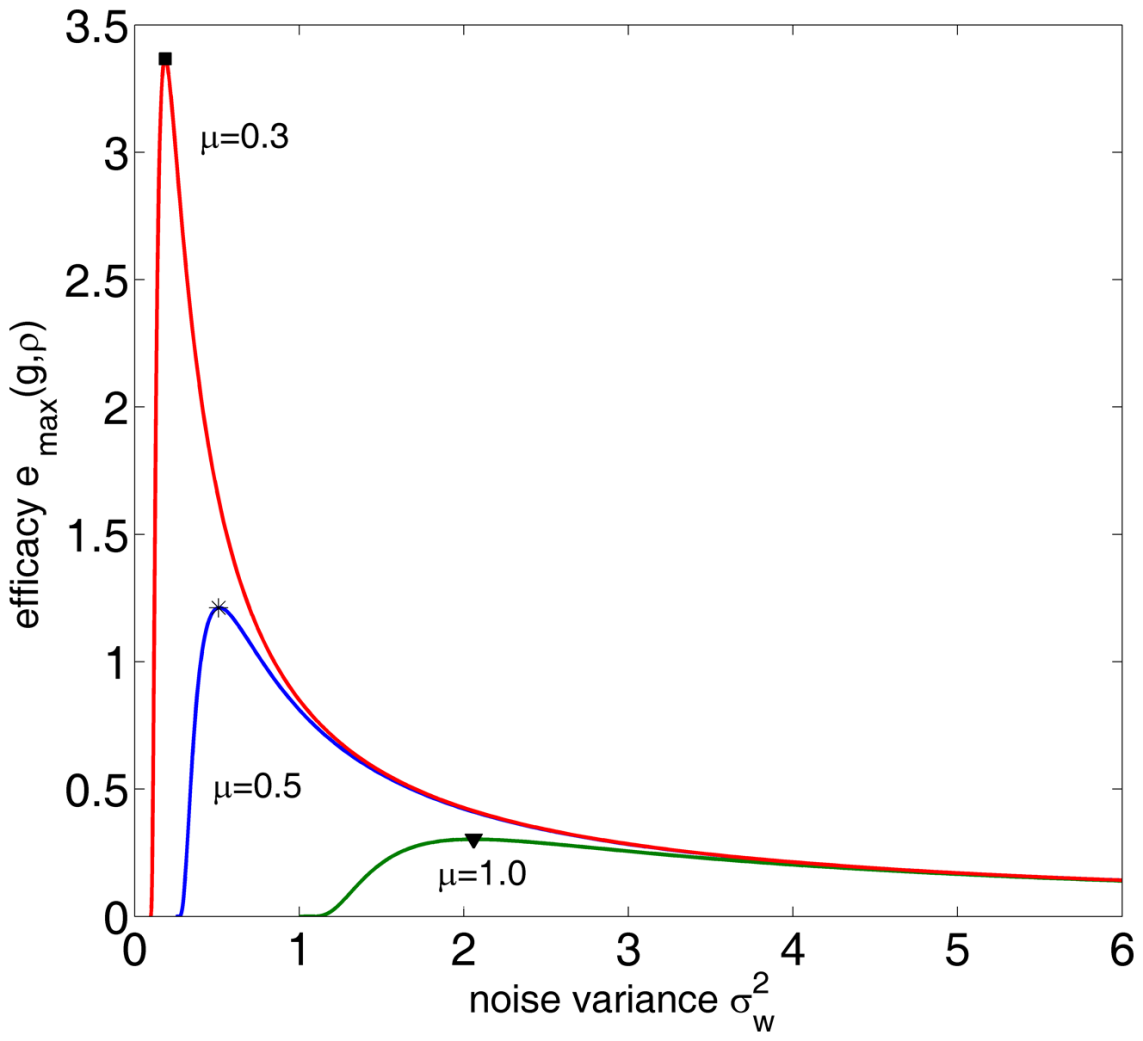
Stochastic resonance of a single threshold neuron. Detection efficacy of 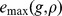 as a function of noise variance 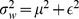 for the correlation coefficient 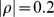 and different values of 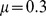 (red), 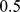 (blue) and 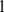 (green). The resonant peaks of 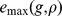 are marked by the square (▪), the star (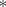) and the down triangle (▾) for 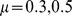 and 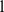, respectively. Here, the transfer function 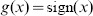, and the noise distribution is Gaussian mixture model of Eq. (33).

We further consider non-scaled Gaussian mixture distribution [5], [47], [48], [60]

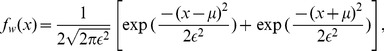
(33)where the variance 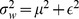 and parameters 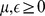. Then, for the signum function 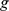 in Eq. (30), the detection efficacy of Eq. (21) can be computed as
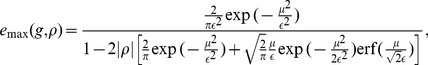
(34)where the error function 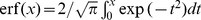. In Fig. 2, for the correlation coefficient 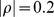 and different values of 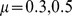 and 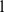, we show the detection efficacy of Eq. (34) as a function of noise variance 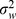. For a given non-zero value 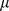 and as 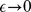, the noise distribution model of Eq. (33) indicates the dichotomous noise [5], [48], [53]. In this situation, as the signal strength 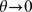 and 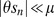, the signum function 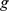 will not change its output whether the signal appears or not. Therefore, the test statistics 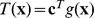 in Eq. (2) will be the same value under hypotheses 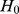 and 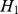, and the detection efficacy 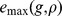 in Eq. (34) starts from zero. This explantation can be also validated by Eq. (34) as 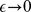 and 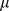 being fixed, as illustrated in Fig. 2. However, it is clearly seen that, upon increasing the noise variance 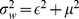 (actually increasing 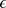), the noise-enhanced detection effect appears. The smaller the parameter 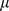 is, the more pronounced the resonant peak of 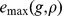 becomes, as shown in Fig. 2.

Next, an interesting problem is that, for scaled noise, can we observe the noise-enhanced detection effect in threshold-based neurons? The answer to this question is affirmative. Here, we will resort to the constructive role of internal noise for improving the performance of an array of threshold neurons. Let 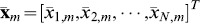 be the vector of 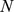 observation components at the 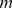-th element of receiving array of 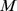 identical neurons. In this observation model, 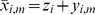 under the hypothesis 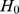. Here, in each neuron element, the 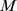 noise terms 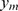 are assumed to be mutually independent with the same PDF 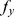 and variance 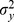. Then, at the observed time 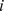, the array outputs are collected as 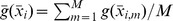, and the test statistics can be reconstructed as 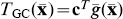 with 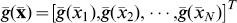. For the colored noise model of Eq. (10) with 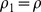 and 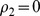, we have
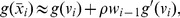
(35)

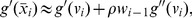
(36)where the composite noise 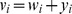 has the convolved distribution 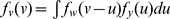. Then, we have expectations
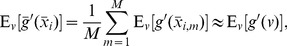
(37)and
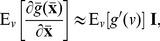
(38)with the operator 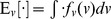. The variance matrix 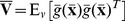 has elements
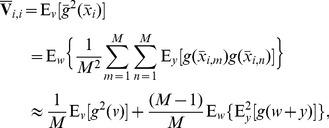
(39)




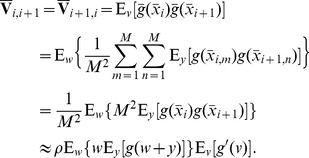
(40)Then, based on Eqs. (38), (39) and (40), the maximum efficacy 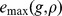 can be computed by Eq. (21) as
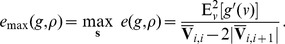
(41)


**Figure 3:**
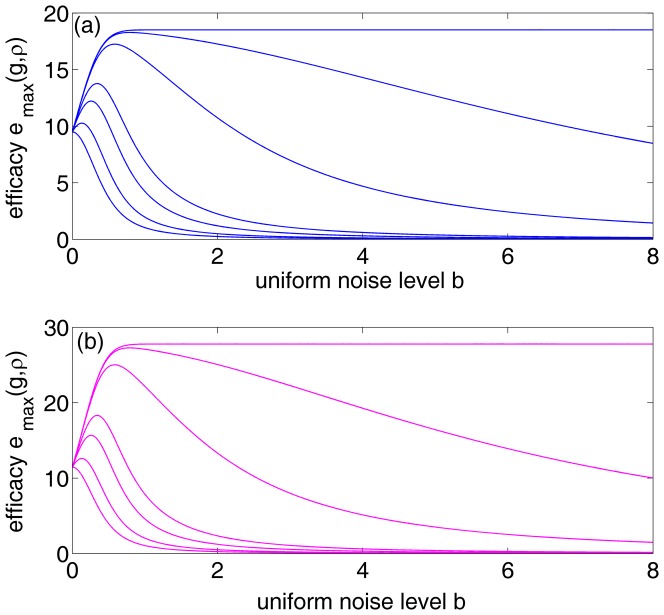
Suprathreshold stochastic resonance in an array of threshold neurons. Detection efficacy 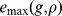 as a function of the internal uniform noise level 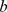 and the neuron array size 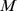. From the bottom upwards, 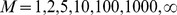. Here, the initial Gaussian noise level 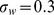, the transfer function 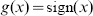, the correlation coefficient (a) 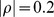 and (b) 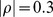.

For instance, we assume the initial Gaussian noise components 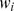 have the distribution of 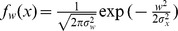 and the given variance 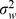. The internal noise components of each neuron is assumed to be the uniform random variable 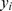 with its distribution 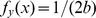 for 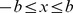 and zero otherwise. The composite random variables 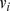 are distributed by
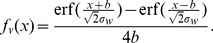
(42)For a given Gaussian noise level 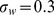, it is shown in Figs. 3 (a) and (b) that the maximum detection efficacy 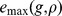 varies as a function of internal uniform noise level 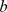 for different array sizes 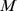 and correlation coefficients 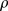. It is noted that, at the uniform noise level 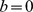, the detection efficacy in Eq. (41) is just the expression of Eq. (32) for a single neuron. Thus, 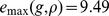 and 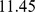 for 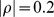 (see Fig. 3 (a)) and 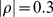 (see Fig. 3 (b)), respectively. By comparing Fig. 3 (a) with Fig. 3 (b), it is seen that the maximum detection efficacy 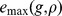 can be further enhanced for a higher value of 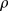. For the array size 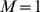 and upon increasing uniform noise level 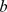, it is seen in Fig. 3 that there is no noise-enhanced effect in a single neuron. However, as 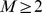, it is illustrated in Fig. 3 that the internal uniform noise can enhance the detection efficacy 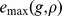, and the noise-enhanced effect does occur. Moreover, as the array size 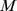 increases, the noise-induced enhancement becomes more visible by adopting an appropriate amount of uniform noise of the neuron array, as shown in Fig. 3. As the detection problem so far is confined to the weak signal with its strength 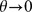 but 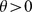, and the response threshold 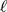 of all neurons is zero, thus Fig. 3 shows the potential capability of suprathreshold stochastic resonance in improving the detection efficacy of a parallel array threshold-based neurons.

## Methods

Under the assumption of weak signals, the Taylor expansion of the function is utilized in Eqs. (4), (5), (11) and (12). The Cauchy-Schwarz inequality is used in Eqs. (7), (9), (23), and (24). The maximum of Rayleigh quotients for a symmetric matrix is calculated in Eqs. (19), (21) and (41).

## Conclusion

In this paper, we study the performance enhancement of threshold-based neurons for detecting weak signals in the presence of colored noise. For a given transfer function, we maximize the detection efficacy by optimally choosing the signal waveform. We prove that colored noise is superior to white noise in enhancing the detection efficacy, at a cost of optimally matching the input signal with the eigenvector of the covariance matrix. Furthermore, we illustrate that, for a single threshold neuron, the possibility of noise-enhanced detection cannot occur in scaled noise, but does appear in a non-scaled Gaussian mixture noise model. Furthermore, for scaled noise, we can test a parallel bundle of neurons with the same response threshold, and recover the positive role of internal noise in enhancing the detection efficacy of the neuron array via the mechanism of suprathreshold stochastic resonance. These results demonstrate that the strategy of exploiting stochastic resonance is still interesting in the case of improving the nonlinear system performance by adding more noise to the signal corrupted by colored noise.

Here, we mainly consider the first-order moving-average noise model of Eq. (10) which is, as we show, amenable to analytical treatment. It is possible to extend the present approach to higher-order moving-average noise models. However, the same analytical treatment maybe no longer feasible. It is also interesting to consider yet other models of colored noise to enhance the detectability of the neuron array. This subject is very promising and currently under study.

It is noted that the detection efficacy of Eqs. (6) and (9) are established under the assumption of weak signal strength 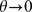. We only consider the first-order Taylor expansion of nonlinearities in Eq. (4), because it makes an analytical treatment possible and the corresponding results are rigorous. In practice, most noise distributions are symmetric and the nonlinear characteristics are odd symmetric about the origin. In this case, we can expand the nonlinearity to the second-order terms. The expectation of the second-order term of Taylor expansion of Eq. (4) vanishes and does not affect the conclusion of this paper. However, for unsymmetrical noise distributions and nonlinearities, the high-order terms of Taylor expansion of Eq. (4) are not exactly zero. For this case, we need to numerically observe the effect of high-order terms on the detector performance. It is interesting to compare the present theoretical results of first-order expansion with the numerical results in the further studies.

We also note that these equations of Eqs. (4)–(9) are the extension of white noise [54], [56]–[58], [60] to the case of colored noise. Then, we consider a model of colored noise allowing for an analytical evaluation of the detection efficacy in Eqs. (6) and (9). The detection efficacy can also be numerically computed to address other models of colored noise, or to explore broader conditions beyond the weak signal limit. As the signal strength 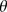 increases, the Taylor expansion of Eq. (4) and the upper bound of Eq. (6) gradually cease to apply. However, based on the present results on weak signal in colored noise, and on [25], [26], [63] on non-weak signal in Gaussian white noise, it can be expected that noise benefit as reported here will persist with colored noise beyond the small-signal limit.
